# Reported Low Uptake of HCV Testing among People Who Inject Drugs in Urban Vietnam

**DOI:** 10.1155/2020/3701379

**Published:** 2020-11-21

**Authors:** Vu Toan Thinh, Do Thi Phuong, Van Dinh Hoa, Le Minh Giang

**Affiliations:** ^1^Center for Research and Training on Substance Use-HIV, Hanoi Medical University, Hanoi 100000, Vietnam; ^2^The Partnership for Health Advancement in Vietnam (HAIVN), Hanoi 100000, Vietnam

## Abstract

**Background:**

HCV testing is an important first step for treatment and prevention, particularly for those who are highly vulnerable to HCV infection such as people who inject drugs (PWID). In settings where direct-acting antiretroviral medicines are becoming more available, limited information exists about who and where to target to increase the prevalence of HCV testing among PWID. This study is aimed at understanding the prevalence of HCV testing uptake and its determinants of medical services and risk behaviors.

**Methods:**

From February 2016 to April 2017, a sample of 509 PWID was interviewed using a structured questionnaire on their history of HCV testing, confirmation, services using in the previous year as well as HCV-related knowledge, and risk behaviors. Multiple logistic regression identified factors associated with ever being tested for HCV before enrollment in the program.

**Results:**

Approximately 33% reported ever testing for HCV. Most cited sources of testing are public hospitals and general clinics (68.9%) and outpatient clinics (18.9%). Having ever tested for HCV was positively associated with accessing health services within the prior 12 months (aOR = 2.25; 95% CI 1.11-4.58), being currently enrolled in a methadone treatment program (aOR = 2.35; 95% CI 1.34-4.08), and/or on ART treatment (aOR = 2.30; 95% CI 1.30-4.08). Those who ever delayed in seeking healthcare services for any reason were less likely to get tested for HCV (aOR = 0.54; 95% CI 0.35-0.84).

**Conclusion:**

HCV testing prevalence is low among PWID in Hanoi despite a very high prevalence of HCV infection. To improve the cascade of HCV testing, it is critical that intervention programs scale up linkages among methadone, outpatient clinics, and HCV services, take steps to reduce stigma and discrimination in both community and, especially, in health care settings, and increase awareness of HCV for PWID by integrating HCV into routine counseling at health care services.

## 1. Introduction

HCV infection among people who inject drugs (PWID) is currently a global public health concern. The prevalence of HCV worldwide among PWID varied from 25% to 90% with a high concentration in Southeast Asia [1–3]. In Vietnam, this prevalence is estimated to have increased from 31% to 97.2% [4–6], and due to the nonexistence of HCV sentinel surveillance, data on the proportion of HCV testing and screening remains limited. In Australia and Greek, the proportions of lifetime HCV testing among PWID occupied up to 90% [7–9] while the corresponding numbers in Scotland [10] and Thailand [11] were much lower (52% and 33%, respectively).

Previous studies showed that appropriate testing has significant benefits both for the individual tested and for public health. While antibody testing plays an important role in detecting exposure to the HCV virus, confirmatory testing is used to detect chronic infection [7]. Without appropriate treatment, HCV infection can, over time, lead to cirrhosis and hepatocellular carcinoma [12, 13]. Particularly, the infected group had a significantly shorter time to develop liver cirrhosis (18.4% vs. 6.1%) and the first hepatic decompensation (3.1% vs. 1.4%) compared to the uninfected group [13]. Once cirrhosis developed, there was a 3-6% annual risk of hepatitis decompensation, and the risk of death in the following year was between 15-20% [14]. Unless people with HCV infection are diagnosed and treated, the number of deaths will continue to increase.

Several studies showed a high percentage of positive antibody-HCV tests; few participants were linked to HCV care [15]. A Tanzania study showed that 37% of PWID testing HCV-seropositive were enrolled in clinical assessment, however, no one was offered HCV treatment. Despite the ongoing risk of transmission, no-repeat testing was provided after registration [16]. Likewise, 59% of PWID in Kentucky reported contacting a healthcare provider within 18 months of a detectable test for HCV infection but only 14% reported seeking HCV treatment, and only 8% reported receiving treatment [17]. This requires future research priorities focus on enhancing HCV prevention, testing, linkage to care, and direct-acting antiretroviral treatment for PWID if efforts to the global elimination of HCV infection are to be successful.

Low HCV uptake among PWID is associated with a number of patient and system-related barriers [9, 11, 18, 19]. Specifically, obtaining an education above secondary school, methadone treatment enrollment, and receiving peer-based education on HCV are positively associated with receiving HCV testing [11]. Being female, older than 25 with an injection history of more than 5 years, and a poly-substance user tend to increase the likelihood of recent HCV testing than others [9]. Otherwise, more than 60% of PWID who have never tested for HCV had a low perception of HCV risk [19]. Other barriers related to stigma and discrimination that may exist within conventional healthcare settings prevent PWID from accessing HCV testing and treatment [11].

In Vietnam, the Ministry of Health recommended screening HCV for PWID but data on HCV testing uptake among this population is not well documented. Previous studies showed that HCV testing uptake was increasing dramatically from 11.2% in 2013 to 19.6% in 2016 [19]. However, no current research activities have been conducted to assess HCV screening and the stages of HCV infection among PWID and to propose effective intervention models for early detection and timely treatment referral in urban settings. Hence, this study was conducted to evaluate the pilot model of providing HCV counseling, testing, and treatment interventions for PWID in Hanoi. This paper seeks to understand the status of HCV testing uptake and associated factors that can inform further intervention to get more PWID to engage in HCV treatment.

## 2. Materials and Methods

### 2.1. Study Design and Sample Size

The Center for Research and Training on Substance Use-HIV (CREATA) performed a cross-sectional study of a cascade of HCV testing from ever getting tested for HCV to viral load counts between February 2016 and April 2017. We conducted a convenient sampling through five community-based organizations of the Vietnam Network of People Who Use Drugs (VNPUDs) network in Hanoi. These trained peers were responsible for outreach PWID who were older than 18 years old, lived in Hanoi at the study enrollment, reported ever injecting at least once in their lifetime, and were voluntarily able to participate. Those who met the criteria were invited to join the study and were referred to the Sexual Health Promotion Clinic (SHP), CREATA where they completed a series of self-report measures. A total of 509 PWID were recruited and completed questionnaires that were then analyzed.

### 2.2. Measures

We ascertained each participant's HCV testing status, demographic characteristics, accessibility to health care service, and risk behavior factors with a semistructured questionnaire.

Self-reported HCV testing cascade included 7 questions: a lifetime history of HCV testing (yes/no), the last time of testing, place of testing (general hospitals, private clinics, outpatient clinics, and rehab centers), testing results (negative, positive, and do not remember), viral load performance (yes/no) and the result (detectable/nondetectable), and HCV treatment (yes/no).

Demographic characteristics included gender (male/female), age group (<30 years old, 30-40 years old, and ≥40 years old), residence (suburban/urban), living arrangement (family members and others), an education level (high school graduated and ungraduated), marital status (married and single/separated/divorced), living with HCV+ individuals (yes/no), occupation status (employed and unemployed), and average income.

The main exposures of interest are factors associated with medical services and risk behaviors: using medical services in the last 12 months (yes/no), ever delayed visiting health care facilities (yes/no), being on methadone treatment (MMT) (yes/no), currently on antiretroviral therapy (ART) (yes/no), HCV knowledge (≤5 and >5 out of 9), ever shared needles with others (yes/no), and ever reused others' needles (yes/no).

### 2.3. Statistical Analysis

We entered data in Epi data 3.1, then cleaned, and analyzed by SAS 13.0 (SAS Institute, Cary, NC, USA). The primary outcome of this analysis was self-reported HCV testing uptake. In the bivariate analysis, the mean was presented for continuous variables, and frequencies and percentages were used for categorical variables. Also, the Chi-square test was applied to determine the difference between HCV testing uptake among demographic characteristics and other associated categorical factors, and a *t*-test was used to determine the difference between HCV testing uptake and continuous variables which were used to build a multivariate model. Variables that showed statistical significance (*p* < 0.2) in the bivariate model and literature review were included in the multivariate model. We used multiple logistic regression to build the final explanatory model.

### 2.4. Ethical Consideration

This study was approved by the Hanoi Medical University Institutional Review Board. All PWID voluntarily participated, and no personal information of participants was collected to ensure confidentiality during study implementation.

## 3. Results

### 3.1. Participant Characteristics

Of 509 PWID who completed the questionnaire, 78.4% were male, 87.8% were 30 years or older, 81.3% lived in urban areas, 81.9% lived with family members, and 82.7% were employed. About 61% of participants graduated from high school or higher. More than 60% reported having a history of incarceration. Participants had an average income of 4.7 million VND (equal to $204 USD), and 13.4% of PWID reported living with HCV positive people (Table 1).

Table 2 showed that about 70% of participants used medical services at least once in the past 12 months, 19% were currently on MMT, 51.6% were on ART treatment, and 48.1% reported that they had ever delayed visiting health care facilities. The average HCV knowledge score was 4.3, and 37% of PWID reported having more than 5 out of a 9-point scale. Around 59% and 65% of participants reported ever sharing needles with others and reusing others' needles, respectively.

### 3.2. HCV Testing Cascade

Regarding the cascade of HCV testing uptake among 509 PWID with 32.2% (164 cases) reported to ever screen for HCV testing at least once in their lifetime. Of those, 92.1% (151 cases) had positive results, but only 18.5% (28 cases) received viral load tests to confirm HCV infection. Twenty-five out of 28 cases (89.3%) were confirmed as HCV infection. No data on receiving the direct-acting antiviral treatments were reported.

Of those who ever tested for HCV, 68.9% were performed at the public general clinics or hospitals, followed by 19% were done at outpatient clinics. The remaining was carried out in private clinics, drug rehab centers, or prison.

### 3.3. Factors Associated with a Lifetime History of HCV Testing

In the bivariate analysis, demographic characteristics were statistically significant association with HCV testing including gender, age group, living arrangement, and marital status. Other factors associated with HCV testing were using medical services in the past 12 months, ever delayed visiting health care facilities, being on MMT and ART, HCV knowledge score, and ever sharing needles with others.

In the multiple logistic model, after controlling for potential confounders, variables positively associated with HCV testing was ever used medical services in the past 12 months (aOR = 2.25; 95% CI: 1.11, 4.58), currently on MMT and ART with aOR = 2.35 (95% CI: 1.34, 4.08) and aOR = 2.30 (95% CI 1.30, 4.08), respectively. The ever delayed in visiting health facilities was a negative association with HCV testing with aOR = 0.54 (95% CI: 0.35, 0.84) (Table 3).

## 4. Discussion

### 4.1. HCV Testing Cascade

Our study found that one-third of PWID in Hanoi ever received HCV testing uptake which is lower in comparison to the HCV prevalence among this population in Vietnam ranged from 31% to 97.2% [4–6]. This proportion of a lifetime history of HCV testing was higher than a study conducted in Hanoi, Thai Nguyen, and Ho Chi Minh city in 2016 that demonstrated 19.6% of PWID had HCV testing [19] but similar to a Thailand study which reported a prevalence of 33% participants ever being tested for HCV. Moreover, findings from the study conducted in heroin users entering substitution treatment in Greece [9] and another study conducted in methadone clinics in China were much higher (83.5% and 78.4%, respectively) [20]. This could be explained by the difference in the study population among studies. Participants from Thailand and our studies accessed PWID from communities while studies in Greece and China enrolled PWID from treatment facilities where resources and supported policies for screening HCV were available [20]. All results emphasize the need for interventions (e.g., HCV screening and testing for all PWID who are admitted to MMT clinics as well as referring HCV+ patients to health facilities for further diagnosis and treatment) to increase HCV testing towards achieving a better cascade of HCV treatment to reduce the risk of HCV transmission.

In our self-reported cascade, of 164 PWID screened for HCV, 92% had anti-HCV positive. This finding is similar to the previous studies that showed that PWID is the most-at-risk group of HCV infection [1–3]. Our finding is found higher than other studies conducted in Vietnam which were from 30-77% in the North [21, 22] and 70-87% in the South [23]. This might be because the differences in study design as well as participants' recruitment strategy. Among those who had positive HCV screening, only 18.5% (28 out of 151) had received a viral load test which shows a large gap from having positive HCV screening to a confirmation test. That meant a small percentage of PWID with a positive HCV screening was offered HCV RNA test. These findings also highlight again the high demand of HCV confirmation test as well as the risk of PWID in infecting others with HCV. A study conducted in the U.S. showed that only two-thirds of patients who had anti-HCV positively were done for the HCV confirmation test due to the fact that patients did not return for follow-up or the facility do not operate RNA test or patients were tested in jails [24]. These are similar in context to studies in other regions of Vietnam as not all facilities which can do HCV screening tests can also do HCV RNA testing, and this deters individuals from returning for a confirmation test. PWID also faced stigma and discrimination in health care settings which prevents them from accessing health care services [25, 26]. This underscores the importance of improving linkages in the healthcare system in general and tracking patients while referring them from a positive screening to a confirmation test since the majority of patients (90%) who received a viral load test were detectable for the virus.

Of those who ever tested for HCV, 69% accessed these tests in general clinics/public hospitals, 18.9% in HIV outpatient clinics (OPC), and the remaining 6.7% in private clinics and jails or 05/06 rehab centers. In contrast, a study conducted in India showed that just 41% accessed HCV testing in government facilities and more than half (55.3%) in private clinics [27]. In Vietnam, the availability of HCV diagnosis and treatment is limited to a few provincial and national hospitals, using a variety of testing reagents [28]. This was also in line with the belief and trend in seeking health care services in central/governmental hospitals in Vietnam [29], especially in Hanoi where many well-known governmental hospitals are located. It is very important that the HCV testing program should be expanded to provincial and district hospitals in order to increase the coverage of HCV testing for high-risk populations.

### 4.2. Factors Associated with HCV Testing Uptake

To our knowledge, this is the first study in Vietnam attempting to understand correlations to HCV testing uptake in PWID. After controlling characteristics in the multiple logistic model, our findings showed that ever used medical services in the past 12 months and currently on MMT or ART treatment were more likely to report a history of HCV testing while ever delayed in visiting health facilities are less likely to have HCV testing. Therefore, it is important to strictly follow the circular on guiding medical examination regularly performed every six months, especially HCV screening and testing among the high-risk populations [30]. In this study, 89% of PWID who had a history of HCV testing used medical services in the past 12 months and were more likely to have HCV testing uptake (aOR = 2.25) while ever delayed in visiting health facilities were less likely to have HCV testing uptake (aOR = 0.54). These findings show the importance of accessing healthcare services towards improving not only health status in general but also HCV testing uptake status. As mentioned above, stigma and discrimination, especially in health care settings, are barriers preventing both PWID and HIV patients to access health care services and remain in care [25, 26, 31, 32]. Efforts towards reducing stigma and discrimination, both in community and in the health care settings, should be made to engage more PWID to access health care services in general and screening for HCV.

Of those who did complete an HCV test, 55% were currently on MMT, and this factor was positively associated with ever receiving an HCV test (aOR = 2.35, 95% CI: 1.36, 4.08). These findings were consistent with previously conducted studies in Thailand, Greece, and China demonstrating the benefits of drug treatment programs, especially opioid and MMT treatment in terms of acquiring health care services [9, 11, 20]. Also, the Vietnamese Ministry of Health guidance on HCV treatment recommends providing anti-HCV tests for high-risk groups, and in this case, we can see benefits from MMT programs that provide counseling as well as linking health care services for PWID. It suggests more effective linkages among HCV services (including testing and treatment), and MMT will increase the numbers of PWID who are diagnosed and enter treatment.

In this study, almost HIV-infected participants who were on ART treatment have accessed HCV testing. After controlling for potential confounders, the odds of having HCV testing uptake were 2.3 times higher in those who are currently on ART treatment compared to those not on ART. This might be because HCV testing is mandatory for people living with HIV/AIDS in Vietnam prior to initiating ART treatment under the national guideline on HIV care and treatment. Additionally, the annually repeated HCV testing will be offered if the previous test is negative [33]. So, it is critical that HCV testing services are provided for those who are most-at-risk populations, like PWID and being HIV+, because HIV/HCV coinfection among PWID is a burgeoning public health problem which threatens to overwhelm clinical care programs.

Some limitations should be considered in our study. First, this was a cross-sectional study, and it is not designed to explain the causality among the outcome of interest and other factors. Yet, it contributes in part to the understanding of determinants of HCV testing among PWID in urban settings. Second, the primary outcome of interest and risk behaviors was self-reported by participants in the last 12 months or a lifetime period, so it may be subjective due to recall bias because of a long time period or social desirability because the aim of the study is to recruit PWID to get free viral load testing, and some PWID may have known their status already and participated to get free viral load testing which may have affected the overall results. Lastly, eligible participants included both active and inactive drug users. In this study, we recruited those who self-reported using drugs in the last 12 months. This might not reflect the population of PWID across all urban settings of Vietnam but we believe that sampling conducted by the five community-based organizations of VNPUDs located in districts with a high density of PWID was representative.

## 5. Conclusion

HCV testing uptake was low among PWID in Hanoi and we found a large gap in HCV testing uptake from positive screening results to confirmation test. Factors positively associated with HCV testing uptake included using medical services in the past 12 months and currently on MMT and ART treatment. The most significant factor negatively associated with HCV testing uptake was ever delayed in visiting health facilities. To improve the cascade of HCV testing, interventions should focus on (1) reducing stigma and discrimination on high-risk groups in community and health care settings, and (2) improving counseling and referral linkages among MMT, OPCs, and HCV services (testing and treatment) for those with a history of drug injection are critical.

## Figures and Tables

**Table 1:** Sociodemographic factors associated with HCV testing uptake.

		Testing for HCV		Total	*p* ^a^
		Yes	No		
		*n* (%)	*n* (%)	*n* (%)	
Gender	Male	142 (86.59)	257 (74.49)	399 (78.39)	0.002
	Female	22 (13.41)	88 (25.51)	110 (21.61)	
Age bracket	Mean ± SD	39.3 ± 5.55	38.2 ± 7.81	38.5 ± 7.17	0.118
	<30	6 (3.66)	56 (16.23)	62 (12.18)	<0.001
	30-40	101 (61.59)	155 (44.93)	256 (50.29)	
	≥40	57 (34.75)	134 (38.84)	191 (37.52)	
Residence	Suburban	32 (19.51)	63 (18.26)	95 (18.66)	0.735
	Urban	132 (80.49)	282 (81.74)	414 (81.34)	
Living arrangement	Others	16 (9.76)	76 (22.03)	92 (18.07)	0.001
	Family	148 (90.24)	269 (77.97)	417 (81.93)	
Educational attainment	Below high school	72 (43.90)	125 (36.23)	197 (38.70)	0.097
	High school and higher	92 (56.10)	220 (63.77)	312 (61.30)	
Marital status	Ever married	37 (22.56)	107 (31.01)	144 (28.29)	0.048
	Never married	127 (77.44)	238 (68.99)	365 (71.71)	
Occupational status	Employed	136 (82.93)	285 (82.61)	421 (82.71)	0.929
	Unemployed	28 (17.07)	60 (17.39)	88 (17.29)	
Average income (million VNĐ^b^)	Mean ± SD	4.4 ± 3.2	4.8 ± 3.5	4.7 ± 3.4	0.1645
Living with HCV individuals	No	140 (85.37)	301 (87.25)	441 (86.64)	0.560
	Yes	24 (14.63)	44 (12.75)	68 (13.36)	
Incarcerated history	No	60 (6.59)	134 (38.84)	194 (38.11)	0.624
	Yes	104 (63.41)	211 (61.16)	315 (61.89)	

^a^Statistical Chi-square differences and *t*-test by HCV testing; ^b^Vietnamese currency ($1 = 23.500VNĐ).

**Table 2:** Health service and other factors associated with HCV testing uptake.

		Testing for HCV		Total	**p** ^a^
		Yes	No		
		**n** (%)	**n** (%)	**n** (%)	
Using medical services in the last 12 months	Yes	146 (89.02)	208 (60.29)	354 (69.55)	<0.001
	No	18 (10.98)	137 (39.71)	155 (30.45)	
Ever delayed visiting health care facilities	Yes	64 (39.02)	181 (52.46)	245 (48.13)	0.005
	No	100 (60.98)	164 (47.54)	264 (51.87)	
Being on methadone treatment	Yes	53 (32.32)	43 (12.57)	96 (18.97)	<0.001
	No	111 (67.68)	299 (87.43)	410 (81.03)	
Currently on ART treatment	Yes	115 (74.68)	134 (40.73)	249 (51.55)	<0.001
	No	39 (25.32)	195 (59.27)	234 (48.45)	
HCV knowledge score	*Mean* ± *SD*	4.82 ± 0.17	4.09 ± 0.12	4.33 ± 0.10	<0.001
	≤5	90 (54.88)	233 (67.54)	323 (63.46)	0.006
	>5	74 (45.12)	112 (32.46)	186 (36.54)	
Ever shared needles with others	Yes	112 (68.29)	187 (54.36)	299 (58.86)	0.003
	No	52 (31.71)	157 (45.64)	209 (41.14)	
Ever reused others' needles	Yes	97 (59.15)	233 (67.54)	330 (64.83)	0.064
	No	67 (40.85)	112 (32.46)	179 (35.17)	

^a^Statistical Chi-square differences and *t*-test by HCV testing.

**Table 3:** Results of the multiple logistic regression analysis with HCV testing uptake.

	**aOR ** ^**b**^	**95% CI**
Used medical services in the last 12 months (vs. no)	**2.25 ** ^**a**^	**1.11-4.58**
Ever delayed in visiting health facilities (vs. no)	**0.54**	**0.35-0.84**
Currently on methadone treatment (vs. no)	**2.35**	**1.36-4.08**
Currently on ART treatment (vs. no)	**2.30**	**1.30-4.08**
Score of HCV knowledge (>5 vs. ≤5)	1.30	0.83-2.05
Ever shared needles with others (vs. no)	1.32	0.76-2.32
Ever reused others' needles (vs. no)	1.04	0.31-1.71

^a^Bolded values indicate differences at the 5% alpha level. ^b^Controlled for gender (male vs. female), age group (<30, 30-40, and more than 40), marital status (ever married vs. never married), and educational attainment (below high school vs. high school and higher).

## Data Availability

The data is available upon request. Please send your request to Vu Toan Thinh (vutoanthinhph@gmail.com).
